# Food Reconstruction Using Isotopic Transferred Signals (FRUITS): A Bayesian Model for Diet Reconstruction

**DOI:** 10.1371/journal.pone.0087436

**Published:** 2014-02-13

**Authors:** Ricardo Fernandes, Andrew R. Millard, Marek Brabec, Marie-Josée Nadeau, Pieter Grootes

**Affiliations:** 1 Leibniz Laboratory for Radiometric Dating and Isotope Research, Christian-Albrechts-Universität zu Kiel, Kiel, Germany; 2 Graduate School "Human Development in Landscapes", Christian-Albrechts-Universität zu Kiel, Kiel, Germany; 3 Department of Archaeology, Durham University, Durham, United Kingdom; 4 Department of Nonlinear Modeling, Institute of Computer Science, Academy of Sciences of the Czech Republic, Prague 8, Czech Republic; 5 Institute for Ecosystem Research, Christian-Albrechts-Universität zu Kiel, Kiel, Germany; Museo Nazionale Preistorico Etnografico ‘L. Pigorini’, Italy

## Abstract

Human and animal diet reconstruction studies that rely on tissue chemical signatures aim at providing estimates on the relative intake of potential food groups. However, several sources of uncertainty need to be considered when handling data. Bayesian mixing models provide a natural platform to handle diverse sources of uncertainty while allowing the user to contribute with prior expert information. The Bayesian mixing model FRUITS (Food Reconstruction Using Isotopic Transferred Signals) was developed for use in diet reconstruction studies. FRUITS incorporates the capability to account for dietary routing, that is, the contribution of different food fractions (e.g. macronutrients) towards a dietary proxy signal measured in the consumer. FRUITS also provides relatively straightforward means for the introduction of prior information on the relative dietary contributions of food groups or food fractions. This type of prior may originate, for instance, from physiological or metabolic studies. FRUITS performance was tested using simulated data and data from a published controlled animal feeding experiment. The feeding experiment data was selected to exemplify the application of the novel capabilities incorporated into FRUITS but also to illustrate some of the aspects that need to be considered when handling data within diet reconstruction studies. FRUITS accurately predicted dietary intakes, and more precise estimates were obtained for dietary scenarios in which expert prior information was included. FRUITS represents a useful tool to achieve accurate and precise food intake estimates in diet reconstruction studies within different scientific fields (e.g. ecology, forensics, archaeology, and dietary physiology).

## Introduction

A research goal common to several different scientific fields (ecology, forensics, archaeology, and dietary physiology) is the quantitative reconstruction of an individual consumer’s diet from the chemical composition of tissues. This is assumed to be possible under the working principle “you are what you eat”, that is, that the chemical signatures of potential food groups are transferred through diet and recorded in consumer tissues [1]. Dietary proxies are here defined as the chemical, isotopic, or elemental, signals measured in consumer tissues at different compositional levels, including single components (e.g., amino acids, fatty acids) [1], [2], [3], [4], [5], [6], [7], [8]. Dietary proxy signals are viewed as representative of a mixture, whose components are the relative contributions of different food groups. Thus, the conceptual framework of a dietary proxy based diet reconstruction is straightforward in principle. Formally, it is based on a particular case of a general mixture decomposition problem [9].

Any diet reconstruction method aims at quantifying unknown mixing contributions relying on chemical signals measured in certain consumer tissues or components and in potential food groups. However, the task is complicated by several factors. The dietary proxy signal will not necessarily reflect the bulk signal of the food groups, but rather that of specific food group fractions (e.g., macronutrients, amino acids, fatty acids). This implies that the concentration of these fractions within a food group needs to be considered. Furthermore, knowledge of dietary routing may also need to be incorporated, since different food fractions may contribute towards a certain dietary proxy signal disproportionately to their occurrence in the diet. Finally, a diet-to-tissue signal offset is commonly observed. In the case of isotopic dietary proxies this offset is often due to isotopic fractionation occurring at different stages of metabolic processes but can also be linked with other aspects such as growth stage, body size, diet quality, nutritional stress, etc [10], [11]. Dietary reconstruction is further complicated by the fact that all quantities involved in the mixture decomposition calculations have non-negligible uncertainties, and these need to be accounted for in any realistic model. In order to be useful, appropriate statistical analysis should produce not only estimates of the contributions of food groups, but also estimates of associated uncertainties. Simultaneously, the uncertainty of the estimates should be reduced as much as possible. Therefore, it is desirable to utilize not only measurement data, but also other sources of *a priori* information in a formalized and unified way.

Existing methods for diet reconstruction include linear mixing models [12]. However, these are only applicable in exactly determined systems (where the number of food groups is the same as the number of dietary proxies plus one), whereas in many research contexts this will often not be the case. An iterative algorithm, such as IsoSource [13] calculates the range of possible solutions based on signal uncertainties defined by the user. However, the IsoSource approach does not acknowledge variability in the isotopic signal of the different food groups nor the uncertainty associated with a diet-to-consumer offset. Work by Parnell et al. [14] and Moore & Semmens [15] cast the diet reconstruction problem in a powerful Bayesian framework. This allowed handling undetermined systems and provided an elegant method for coping with different sources of uncertainty. However, these methods do not account for possible dietary routing mechanisms. Recent research has demonstrated the importance of considering macronutrient dietary routing, expressed through a fraction weight contribution, to provide more accurate dietary estimates [16]. Existing models also do not provide a simple method allowing the user to input diverse sources of prior information. In this respect, the possibility of introducing prior information establishing relationships on the intake of food groups or food fractions would be very useful. Examples of relevant prior information include knowledge of relative consumption of different food groups, acceptable intake ranges of certain food fractions, etc.

Here, the novel Bayesian mixing model FRUITS (Food Reconstruction Using Isotopic Transferred Signals) is introduced. FRUITS is capable of handling dietary routing and provides a platform that simplifies the incorporation of *a priori* information, including information from ecological, archaeological, biochemical or physiological sources. A user-friendly application of FRUITS is available for download (https://sourceforge.net/projects/fruits/files/) as Open Source software.

## Methods

### 2.1 Main Model: Intake of Food Groups

The formalization of mixture for diet reconstruction is based on equation (1). The main goal of a diet reconstruction exercise is to determine the contribution (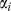) of each *i*-th food group towards a consumer diet. This is achieved by measuring isotopic or elemental signals (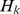) in consumer tissues (e.g. bone bioapatite, bone bulk collagen, bone collagen single amino acids, etc.). Consumer signals result from the mixing of the *k*-th isotopic or elemental signal (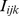) measured in the *j*-th food fraction (e.g. protein, carbohydrates, lipids, single amino acids) of each *i*-th food group (e.g. plant, animal, fish). The model also accounts for a possible diet-to-tissue offset (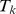) that may result, for instance, from isotopic fractionation during tissue building. Dietary signal contribution is weighed by the concentration (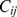) of the *j*-th food fraction (e.g. macronutrients) in the *i*-th food group. Finally, in case of a routed model the weight parameter (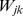) establishes the contribution of the *j*-th food fraction towards the *k*-th consumer signal.

The model formulated in equation (1) is similar to already existing models [14]. However, the expansion introduced by the inclusion of the weight contribution (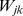) of different food fractions towards a consumer signal allows for the use of dietary proxies in which dietary routing needs to be taken into account.
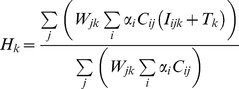
(1)where:


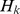
 *k*-th dietary proxy signal measured in the consumer, modelled as a normal distribution, 
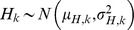
 with 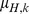 representing the average value and 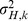 the associated variance.


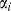
 dietary proportion of the *i*-th food group.
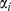
’s are unknown, their estimation and estimation of their uncertainties represents the ultimate analytical goal. Physical restrictions apply: 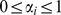 for 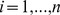 and 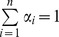 with 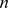 representing the number of food groups.


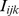
 isotopic or elemental signal from the *i*-th food group, the *j*-th food fraction, and associated with the *k*-th dietary proxy. Due to the presence of measurement errors (and inter-individual heterogeneity), it is assumed to behave as a random variable which is modelled by a normal distribution, 
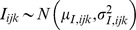
.


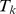
 diet-to-tissue offset for the *k*-th dietary proxy signal. Modelled as a normal variable, 
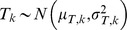
.


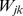
 weight contribution of the *j*-th food fraction in forming the *k*-th target signal. Modelled as a normal variable, 
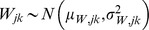
.


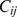
 concentration of the *j*-th fraction in *i*-th food group. Modelled as a normal variable, 
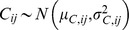
.

Equation (1), through the weight parameter 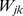, accounts for dietary routing of food fractions, a capability absent in previous approaches, adding an additional layer of decomposition in the mixture. The general estimation approach for model (1) is based on the Bayesian paradigm [17]. In a Bayesian analysis prior distributions, or simply priors, of model parameters are defined by the user. Parameter posterior distributions are determined combining user-defined priors and a likelihood function based on observed data and the probability model (1) [18]. For the unknown parameters uninformative or mildly informative prior distributions can be employed. A standard option for 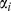
’s is the use of a Dirichlet prior, which is a generalization of the Beta distribution, with unit hyperparameters [19].

FRUITS graphical interface generates a BUGS (Bayesian inference Using Gibbs Sampling) script code, this script is then automatically executed using the OpenBUGS software package. OpenBUGS is a well-established framework for analysing Bayesian probability models [20]. Model computations are based on Markov chain Monte Carlo (MCMC) simulations yielding, upon convergence of the sampler, a posterior distribution [21]. Model output consists of credible intervals and posterior probability distributions. When necessary, OpenBUGS users can easily obtain additional summary outputs in addition to those already provided by FRUITS.

### 2.2 Additional Model Estimates

In addition to estimates on the intake of food groups (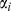) FRUITS also provides estimates of other quantities, and associated uncertainties, of potential interest. These estimates can be of use in different situations, including: providing useful information to address specific research questions, assessing model performance, and extending possibilities for the inclusion of expert information.

Two other estimates provided by FRUITS are the relative contributions of the *j*-th food fraction towards the entire diet (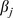), and the relative contribution of the *i*-th food group towards the *k*-th dietary proxy signal (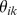).

Expression (2) represents a simple weighed average, through fraction concentration (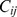), of food group intake (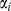). This provides an estimate on the relative contribution of each *j*-th food fraction towards the total dietary intake.
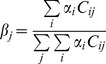
(2)


Prior constraints on 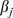 can also be applied, for instance, when restrictions on the relative intake of macronutrients apply. This type of prior information will typically originate from metabolic and physiological studies. The incorporation of these types of priors should improve the overall precision of model estimates.

Estimates on the relative contribution of the *i*-th food group towards a *k*-th dietary proxy signal are determined using expression (3).
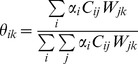
(3)


Estimates of 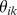 can be of use, for instance, in providing radiocarbon dating corrections for cases in which human dietary radiocarbon reservoir effects are observed. Given that aquatic food groups are often depleted in ^14^C, older than expected human bone collagen radiocarbon ages are observed when an individual had a diet that includes aquatic food groups. Human dietary reservoir effects are exemplified in Fernandes et al. [22] which also includes a first application of FRUITS in an archaeological context. Estimates of 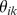 associated with the dietary proxy δ^13^C_coll_ (δ^13^C measured in human bone collagen) can be used to quantify the amount of carbon originating from aquatic food groups.

### 2.3 Adding Prior Information

Since diet decomposition from isotopic or elemental data involves several sources of uncertainty translating into uncertainty of the resulting food group proportions (uncertainties of the posterior 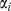 estimates), all available sources of prior information should be explored efficiently. This is not entirely straightforward and might include heterogeneous data and information sources other than signal measurements. For example, it is imperative to build into the model natural constraints on proportional intakes of food groups. These constraints are not always as simple as the sum-to-one restrictions on 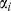
’s or interval (feasibility) restrictions on concentrations and offset factors.

Previous models have included the possibility of providing informative Dirichlet priors on 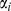. A simple way to do this is for the user to specify a beta distribution for each proportion. The shape parameters 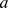 and 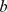 of a beta distribution can be determined from mean (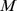) and standard deviation values (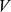) using Equations (4) and (5) [19].
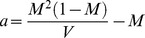
(4)




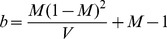
(5)


However, the specification of the parameters presents difficulties not present, for instance, in the specification of parameters of a normal distribution. In a normal distribution, 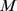 and 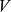 specify completely different aspects of the distribution (location and variability around the location) with the distribution shape remaining the same, irrespective of parameter combination. In the case of a beta distribution 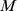 and 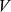 (or, 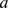 and 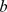) are tied not only to location and variability but, in a complex manner, to higher order moments and indeed, the whole shape of the distribution. Thus, beta distributions having different 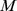 and 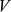 combinations will present considerably different shapes, some of which will not correspond to realistic situations.

In FRUITS a simple approach has been developed for incorporating *a priori* constraints of non-standard types into the expanded version of model (1). The alternative offered by FRUITS is to incorporate prior expert information through the building of algebraic expressions 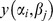 that express relationships of equality or inequality between different 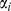
’s and 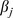
’s (e.g. when prior knowledge allows to impose that certain food groups or food fractions contribute more than others). Such an expression is constructed so that if it represents a required equality it evaluates to zero when that equality occurs, and if it represents a required inequality it evaluates to a positive number when the inequality holds.

To link a relationship of equality into the BUGS model a parameter 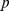 (6) is assigned a likelihood which is normally distributed with the mean given by 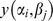 and a fixed uncertainty 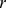. The actual value of 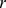 depends on user input and is chosen such that is much smaller than reported uncertainties in the 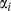
’s and 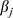
’s.
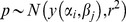
(6)


The equality constraint is imposed by having an ‘observed’ value of zero for 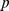.

To link an inequality relationship into the BUGS model we assign parameter 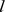 (7) a Bernoulli distributed likelihood 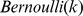 where 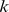 is a Heaviside function 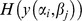 which provides a value of one or zero depending on whether 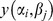 is positive or negative. The inequality constraint is then imposed by having the ‘observed’ value of one for 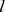.
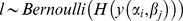
(7)


Depending on the choice of 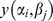, this could provide strong priors and the user should take some caution in verifying that the proposed model corresponds to a realistic situation.

## Assessing FRUITS Performance

### 3.1 Testing FRUITS using Simulated Data

Although FRUITS is capable of handling dietary routing it can also be used to provide dietary estimates in non-routed models. This is here illustrated using simulated data. Table 1 shows protein δ^15^N isotopic values for three different food groups and the dietary intake percentage of a hypothetical consumer. A value of 3‰ was taken for the diet-to-consumer tissue isotopic offset. For the isotopic values and intake amounts reported in Table 1 this implies a consumer tissue δ^15^N value of 6.6‰.

**Table 1:** Protein isotopic values of food groups and relative dietary intakes for a simulated consumer.

Food group	δ^15^N (‰)	Intake (%)	Estimated intake (%)
Plant	2	70	69±7
Animal	6	20	21±12
Fish	10	10	10±6

Estimates of protein intakes provided by FRUITS are listed together with associated standard deviation.

A FRUITS model (see also FRUITS file S1) to estimate protein contributions was defined with three food groups (plant, animal, and fish), one food fraction (protein), and one dietary proxy (δ^15^N). In this model corresponding to a simulated scenario the ‘measured’ consumer δ^15^N value was set at 6.6±0.2‰. A non-routed dietary proxy can easily be defined in FRUITS by assigning a zero contribution to non-relevant food fractions. In the example presented here only one dietary proxy and one food fraction are considered and as such FRUITS assigns by convention the value 100 to the weight contribution of protein towards the δ^15^N signal. For the model parameter diet-to-consumer tissue offset the value of 3‰ was taken as mentioned previously. Model food values were as listed in Table 1. Given that the goal of the exercise is to estimate the protein contribution of the different food groups the model parameter protein concentration was set at 100 for all food groups.

**Figure 1:**
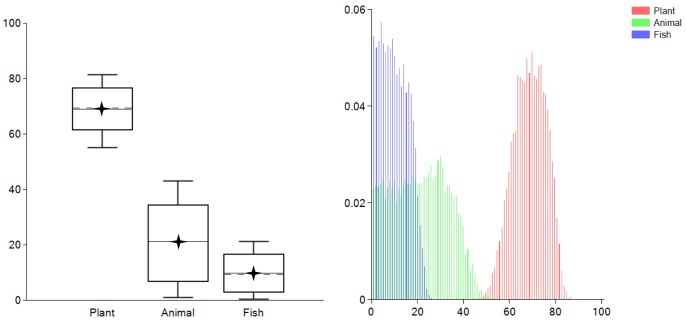
Model output for simulated data (credible intervals on the left and probability distributions on the right). Boxes represent a 68% credible interval (corresponding to the 16th and 84th percentiles) while the whiskers represent a 95% credible interval (corresponding to the 2.5th and 97.5th percentiles). The horizontal continuous line represents the estimated mean while the horizontal dashed line represents the estimated median (50th percentile). Star symbols represent the dietary intake amounts used to simulate data.

Model estimates are represented in Figure 1 as probability distributions and box and whisker plots. Table 1 lists the average estimates generated by FRUITS of protein contributions towards consumer diet. Comparison of intake values used to simulate consumer isotopic value and FRUITS average estimates shows an almost perfect agreement (Table 1).

### 3.2 Testing FRUITS on a Real Case Study (Hare et al. 1991)

Data from the published study by Hare et al. [6], a controlled animal feeding experiment, was chosen to test FRUITS. This case study serves to illustrate how the novel capabilities provided by FRUITS can be employed and also exemplifies some of the issues that need to be considered when handling data within a diet reconstruction study. Hare et al. investigated diet-to-tissue isotopic offsets in groups of pigs raised on well-defined diets. Although FRUITS is oriented towards individual consumer diet reconstruction, group estimates can also be made by using average group values and associated uncertainties. The data listed here refers to a dietary group consisting of 10 pigs raised predominately on C4-based food groups. Different dietary proxies (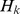) were employed in the study, including measurements on several collagen amino acids. Here, only three dietary proxies are considered, δ^13^C (13Ccoll) and δ^15^N (15Ncoll) measured in pig bone bulk collagen, and δ^13^C measured in the amino acid glutamate isolated from bone collagen (13Cglu). Table 2 lists the isotopic values associated with each dietary proxy. The experimental uncertainty of isotopic measurements was 0.5‰, since group average uncertainty was not reported the experimental uncertainty was used as model input.

**Table 2:** Average (10 pigs) isotopic values and associated uncertainties of dietary proxies.

Dietary proxy	13Ccoll	15Ncoll	13Cglu
Signal value (‰)	−9.2(0.5)	5.5(0.5)	−5.5(0.5)

Dietary proxies: bone bulk collagen δ^13^C (13Ccoll), bone bulk collagen δ^15^N (15Ncoll), and collagen-extracted glutamate δ^13^C (13Cglu).

Five main food groups were listed in the experiment (Table 3). Macronutrient (protein, carbohydrates, and lipids) composition was not reported and reference values were obtained from the databases of the National Nutrient Database for Standard Reference of the United States Department of Agriculture and available data on feed composition according to the specifications of the National Grain and Feed Association [23], [24]. The concentration of the different nutrients (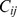) is expressed as normalized dry weight (wt %), the carbohydrate weight contribution (Carbs) includes only digestible carbohydrates (fibre contribution was subtracted), and energy represents the added weight contribution of lipids and carbohydrates.

**Table 3:** Main food groups with total and carbon-only normalized dry weight composition of macronutrients.

Food group	Soybean meal	Barley grain	Alfalfa	Ground corn	Corn gluten meal
Code	Soybean	Barley	Alfalfa	Corn	Gluten
Intake (wt %)	0	0	0	69	31
Protein (wt %)	62	15	30	12	72
Lipids (wt %)	1	3	5	6	3
Carbs (wt %)	37	82	64	83	25
Energy (wt %)	38	85	70	88	28
Protein (wtC %)	32(2.5)	8(2.5)	16(2.5)	6(2.5)	38(2.5)
Lipids (wtC %)	1(2.5)	2(2.5)	4(2.5)	4(2.5)	2(2.5)
Carbs (wtC %)	17(2.5)	36(2.5)	29(2.5)	37(2.5)	11(2.5)
Energy (wtC %)	18(2.5)	38(2.5)	33(2.5)	41(2.5)	13(2.5)

Energy refers to the added contribution of carbohydrates and lipids. Values in parentheses represent the uncertainty assigned to carbon composition. Reported intake values are expressed as normalized dry weight contributions.

When defining the composition of food groups, the relevant quantity is the elemental concentration within each food group fraction. Thus, the carbon weight composition (wtC %) for each food fraction was determined using reference macronutrient carbon contents (protein 52.4%, carbohydrates 44.4% and lipids 76.8%) [25]. A conservative absolute uncertainty was associated with the carbon content of each food fraction (2.5%). FRUITS estimates are based on the relative fraction composition of each food group relative to a common base reference (in this example food dry weight). Given that carbon and nitrogen contents of the protein fraction are proportional, it is not necessary to estimate the nitrogen composition of each food group and the value reported for carbon can be employed instead.

Table 4 lists the diet-to-tissue isotopic offset values (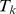) and the weight contributions (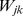) of different food fractions towards a dietary proxy signal defined relying on data from controlled animal feeding experiments on omnivorous mammals [8], [26], [27], [28], [29]. In omnivorous mammals, the 13Ccoll signal is determined from relatively fixed contributions of dietary protein and energy. These contributions were established in the study by Fernandes et al. [16] that analysed data collected from several feeding experiments on omnivorous mammals. Statistical analysis indicated a diet-to-collagen δ^13^C offset of 4.8±0.2 ‰. However, a more conservative uncertainty (0.5‰) was used here to account for possible body size effects as reported in previous experiments which analysed not bone but teeth [10]. Statistical analysis also provided a weight signal contribution from dietary protein of 74±4% and the remainder 26% from dietary energy (carbohydrates and lipids) [16]. For the 15Ncoll dietary proxy, analysis of data collected from feeding experiments on omnivorous mammals, which includes experiments on the effects of dietary stress, gives a δ^15^N diet to bulk collagen offset of 3.6±1.2‰ [2], [6], [8], [11], [30]. For the 13Cglu proxy, the study by Howland et al. [8] demonstrated an excellent correlation (R^2^ = 0.96) between the bone collagen glutamate δ^13^C signal and the δ^13^C signal of the scrambled dietary mix. Estimated δ^13^C bulk diet to bone collagen glutamate offset was 9.2±1.8‰.

**Table 4:** Diet-to-tissue offset and weight contribution of the different food fractions towards a dietary proxy signal.

Dietary proxy	Offset	Weight contribution		
		Bulk	Protein	Energy
13Ccoll	4.8(0.5)	0(−)	74(4)	26(−)
15Ncoll	3.6(1.2)	0(−)	100(−)	0(−)
13Cglu	9.2(1.8)	100(−)	0(−)	0(−)

Values in parentheses represent uncertainty. When a dash sign is used this indicates that the uncertainty was considered negligible.

The study by Hare et al. reported only bulk isotopic values of the different food groups. These values are relevant for the dietary proxy 13Cglu which provides a signal for the scrambled dietary mix and for the dietary proxy 15Ncoll since the only source of dietary nitrogen is protein. However, for the 13Ccoll proxy, dietary routing needs to be taken into account and the isotopic values of the food group fractions protein and energy are necessary. These can be estimated by considering reported isotopic offset values in cereal grains between the bulk δ^13^C value and protein (ca. −2‰) and carbohydrate (ca. +0.5‰) δ^13^C values [31]. Lipids were not taken into account since these represent a minor contribution to the listed food groups. Table 5 lists the isotopic values (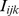) of the different food groups’ fractions for the proposed dietary scenario.

**Table 5:** Fraction isotopic values for the different food groups.

Code	Fraction	13Ccoll	15Ncoll	13Cglu
Soybean	Bulk	–	–	−24.0(0.5)
Soybean	Protein	−26.0(0.9)	−0.1(0.5)	–
Soybean	Energy	−23.5(0.9)	–	–
Barley	Bulk	–	–	−25.3(0.5)
Barley	Protein	−27.3(0.9)	2.6(0.5)	–
Barley	Energy	−24.8(0.9)	–	–
Alfalfa	Bulk	–	–	−26.0(0.5)
Alfalfa	Protein	−28.0(0.9)	0.7(0.5)	–
Alfalfa	Energy	−25.5(0.9)	–	–
Corn	Bulk	–	–	−11.3(0.5)
Corn	Protein	−13.3(0.9)	6.3(0.5)	–
Corn	Energy	−10.8(0.9)	–	–
Gluten	Bulk	–	–	−13.2(0.5)
Gluten	Protein	−15.2(0.9)	3.0(0.5)	–
Gluten	Energy	−12.7(0.9)	–	–

Values in parentheses represent associated uncertainty. When a dash sign is used this indicates no contribution.

### 3.3 FRUITS Results for Hare et al. (1991) Data

**Figure 2:**
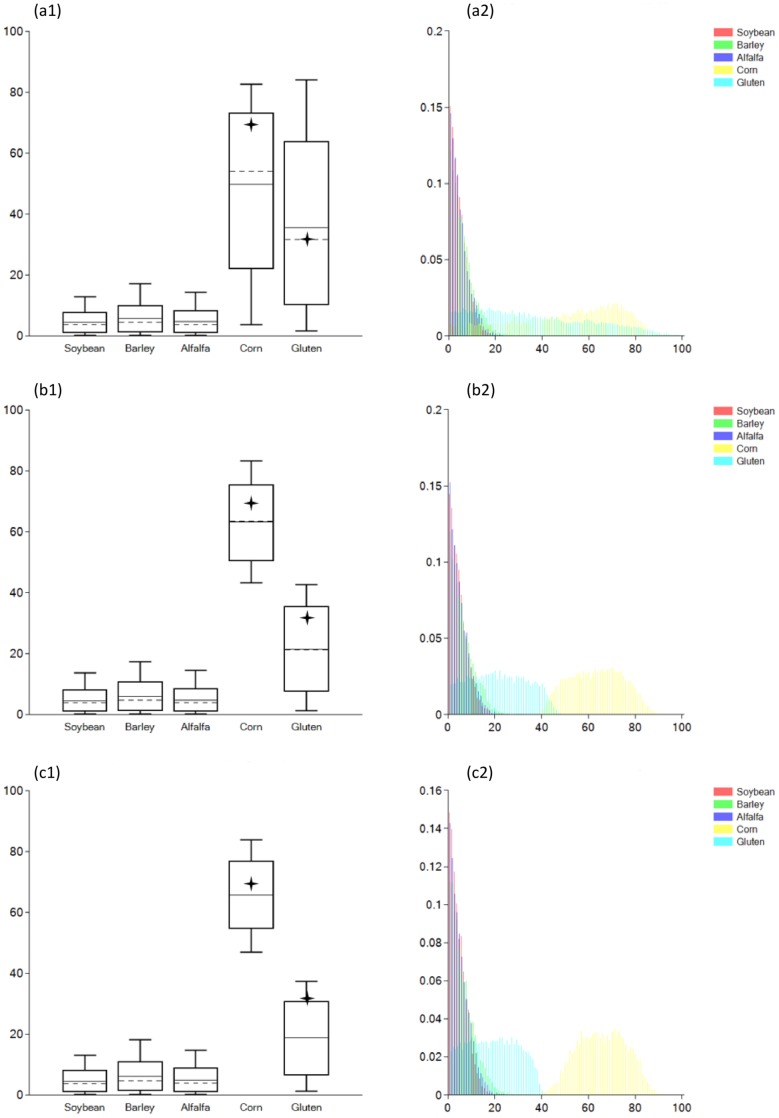
Model output using data from Hare et [6] (credible intervals on the left and probability distributions on the right) for proposed dietary scenarios (a), (b), and (c). Boxes represent a 68% credible interval (corresponding to the 16th and 84th percentiles) while the whiskers represent a 95% credible interval (corresponding to the 2.5th and 97.5th percentiles). The horizontal dashed line represents the estimated mean while the horizontal discontinuous line represents the estimated median (50th percentile). Star symbols represent the actual dietary intake amounts of Corn and Gluten.

A particular dietary scenario is defined by the combination of selected food groups, concentrations of food groups’ fractions and associated isotopic values, diet-to-tissue isotopic offsets, fraction weight contributions, and prior information. Interpretation of generated model results has to be framed within the selected dietary scenario. Three main scenarios (a, b, and c) were here considered and model estimates of food groups intake, expressed as credible intervals and probability distributions, associated with each scenario are represented in Figure 2.

#### 3.3.1 Dietary scenario (a)

In scenario (a) no additional prior information was included and data input was as listed in Tables 2, 3, 4, and 5 (see also FRUITS file S2). The results are expressed as credible intervals in Figure 2 (a1), and as probability distributions in Figure 2 (a2). Boxes represent a 68% credible interval (corresponding to the 16th and 84th percentiles) while the whiskers represent a 95% credible interval (corresponding to the 2.5th and 97.5th percentiles). The results show that the proposed scenario correctly predicts that C4-type food groups (Corn and Gluten) were being consumed. However, although the results show that Corn has higher mean and median estimated intake versus Gluten, the 95% credible interval range is very wide and the estimates on the intake of Corn versus Gluten are statistically indistinguishable. In part this is due to the conservative assumptions adopted for scenario (a), however, the principal cause is that Gluten and Corn have similar isotopic values (Table 5) and the dietary proxies chosen cannot separate the contributions of the two food groups.

#### 3.3.2. Dietary scenario (b)

In scenario (b) data input was the same as in scenario (a), except that a prior was added imposing that the intake of Corn is larger than the intake of Gluten (see also FRUITS file S3). This is represented in FRUITS notation as:
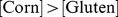
(8)


FRUITS output is represented in Figure 2 (b1) and Figure 2 (b2). The model still accurately predicts that the major food group contributions are from Corn and Gluten, however, in comparison with scenario (a) the credible intervals and probability distributions are now significantly narrower.

#### 3.3.3 Dietary scenario (c)

In scenario (c) data input was the same as in scenario (a). Here, a different prior from scenario (b) is included establishing the acceptable intake of dietary protein (see also FRUITS file S4). A minimum intake of protein is required to perform different body functions like tissue building [32], [33], [34], while excessive protein intake may result in adverse health conditions such as “rabbit starvation syndrome” [35], [36], [37], [38]. Given the similarities between human and pig metabolism, the reference protein intake value for humans was here considered. The acceptable reference range for protein intake as established by the Institute of Medicine of the U.S. National Academy of Sciences is between 10 and 35% of total calorie intake [39]. To express calorie intake as carbon intake the energetic and carbon content of the different macronutrients needs to be considered: carbohydrates (4 kcal/g; 44.4 C %), protein (4 kcal/g; 52.4 C %), and lipids (9 kcal/g; 76.9 C %) [25], [39]. Using these values the energy to carbon content ratios can easily be determined and are approximately: carbohydrates 9.0 kcal/g C, protein 7.6 kcal/g C, and lipids 11.7 kcal/g C where g C represents grams of carbon. Thus, the energy to carbon content ratio is broadly similar for the different macronutrients and here considered effectively the same. However, to account for the ratio variations, a conservative range of protein intake between 5 and 40% of total macronutrients was taken. This is expressed in FRUITS notation as:
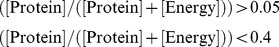
(9)


The output provided by FRUITS is represented in Figure 2 (c1) and Figure 2 (c2). Again the model accurately predicts the contributions of Corn and Gluten, although, the intake of Gluten is not now within the 68% credible interval but is within the 95% credible interval. Compared with scenario (b) the credible intervals are now even more precise.

## Discussion

The application of FRUITS has been illustrated through simulated data and a real case study relying on published data from a controlled animal feeding experiment. The selected case study demonstrates the use of the novel model capabilities introduced by FRUITS and also some of the aspects that need to be considered when handling data in diet reconstruction studies. These include the need to provide estimates on concentration and isotopic values of the relevant fractions in potential food groups. Ideally, such quantities should be measured directly on available food groups. However, in certain research contexts this data might not be directly available. Relevant examples are frequent in archaeological studies for which individual diet reconstruction is of particular interest. In archaeological studies, the establishment of an isotopic baseline often relies on data from food remains (e.g., animal bone collagen, charred grains) that do not necessarily match the isotopic values of edible food fractions (e.g., meat protein, plant carbohydrates). Using knowledge of typical isotopic offsets between edible and recovered food remains, it is possible to provide conservative estimates of food fractions isotopic values, which can easily be handled within FRUITS. For the presented real case study, the use of conservative uncertainties for the different model parameters defined a simple dietary scenario (a) that accurately identified the two main food groups that were consumed. The case study also illustrates the significant improvement brought by the introduction of additional prior information establishing quantitative relationships on the relative intake of food groups, scenario (b), and of food fractions, scenario (c). Scenario (c) is of particular interest, as it demonstrates how information obtained from metabolic studies can be incorporated into a diet reconstruction model. The use of this type of priors can greatly increase the precision of generated intake estimates.

FRUITS is capable of providing accurate and precise estimates on food intake. Nevertheless, this requires that several conditions are met, namely, the proposed dietary scenarios approximate the real scenario, the identified food groups should have significantly different chemical signatures, and the selected dietary proxies are in sufficient number to offer the possibility of separating the contributions of the different food groups. This implies that there is a good knowledge of the accessible individual foods and of their corresponding fraction composition and chemical signatures. In some instances, individual food types having similar fraction characteristics can be aggregated into food groups and associated uncertainties should reflect this aggregation process. The number of dietary proxies, preferentially associated with distinct dietary routing mechanisms, should increase as the number of potential food groups also becomes larger. As shown in the selected real case study, model estimates benefit greatly from the use of prior information on expected ranges or relative intakes of food groups or fractions. However, the user should be certain that the priors chosen are based on well-founded knowledge. This can include research results from metabolic or physiological studies. Finally, to test the reliability of generated results it is important that model outputs corresponding to different dietary scenarios are compared to assess the degree of model sensitivity and robustness.

The model used by FRUITS could be further elaborated to allow diet-to-tissue offsets which vary by food group or food fraction (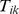 or 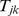). There is some evidence that diet-to-tissue offsets can vary with the composition of food items [40], [41], and this would therefore be the next logical step in development of statistical models for dietary reconstruction. As the output given by FRUITS includes the full BUGS model code used to generate model estimates, users familiar with BUGS can easily modify the model and include additional parameters not available through the graphical interface. Other developments, might include, among others, the addition of a general error term, additional priors, etc. Through user feedback it will be possible to develop an ever more user friendly and sophisticated application.

## Conclusions

FRUITS is a novel Bayesian mixing model that efficiently handles knowledge on dietary routing mechanisms and provides a platform for the simple introduction of expert prior information to arrive at an accurate diet reconstruction, complete with uncertainty estimates, based on chemical and isotopic dietary proxies.

Tested on a real case study, a published controlled animal feeding experiment, FRUITS was capable of accurately predicting consumed food groups. Different dietary scenarios were tested, and in scenarios where expert prior knowledge was introduced a significant improvement in estimate precision was observed.

It is hoped that FRUITS will become a useful tool in diet reconstruction studies associated with different scientific fields (e.g. ecology, forensics, archaeology, and dietary physiology).

## Supporting Information

FRUITS File S1 **FRUITS file corresponding to simulated data.**
(FRT)

FRUITS File S2 **FRUITS file corresponding to dietary scenario (a) using data from Hare et al. (1991).**
(FRT)

FRUITS File S3 **FRUITS file corresponding to dietary scenario (b) using data from Hare et al. (1991).**
(FRT)

FRUITS File S4 **FRUITS file corresponding to dietary scenario (c) using data from Hare et al. (1991).**
(FRT)
